# Enhancing Sugarcane Yield and Weed Control Sustainability with Degradable Film Mulching

**DOI:** 10.3390/plants14162521

**Published:** 2025-08-13

**Authors:** Xin Yuan, Rudan Li, Guolei Tang, Shaolin Yang, Jun Deng

**Affiliations:** 1 Sugarcane Research Institute, Yunnan Academy of Agricultural Sciences, Kaiyuan 661699, China; m18214394679@163.com (X.Y.); lirudan@126.com (R.L.); kytgl@163.com (G.T.); cbttxsysl@foxmail.com (S.Y.); 2 Yunnan Engineering Research Center of Sugar Industry, Kaiyuan 661699, China

**Keywords:** sugarcane yield, weeds, biodegradable plastic film, film mulching

## Abstract

A two-year field study evaluated biodegradable plastic film (BPF; thicknesses: 0.006, 0.008, and 0.010 mm) versus polyethylene film (PE; 0.010 mm) and no-mulch control on sugarcane yield and weed suppression. Key results demonstrated that 0.010 mm BPF significantly enhanced sugarcane emergence (*CV* = 5.07% in ratoon), reduced weed biomass by 70%, and increased perennial yield by 3.83% (+5.6 t ha^−1^), while PE film decreased yield by 3.80%. Regression analysis identified the effective stem number, plant height, and stem diameter as primary yield predictors (*R*^2^ = 0.996). Logistic models revealed that film mulching duration >119 days was critical for achieving high yields (>122.2 t ha^−1^) and sustained weed control (*R*^2^ = 0.81). These findings establish 0.010 mm BPF as an optimal sustainable alternative to PE film for enhancing sugarcane productivity.

## 1. Introduction

As the world’s largest agricultural producer and consumer of key agricultural products such as maize and other water-intensive staples, China’s pursuit of sustainable agriculture faces intensified pressures in dryland regions. These areas grapple with increasing climate volatility and acute water scarcity, threatening crop productivity and resource resilience, especially for crops where water conservation strategies, including plastic film mulching (PFM), are critical [1,2,3]. To address these issues, PFM emerges as a widely adopted and promising solution, demonstrably enhancing soil water content retention and improving water-use efficiency [4,5,6]. Furthermore, this practice provides a stable temperature for crop growth, fosters early germination and overall crop development [7]. In light of the paramount importance of food security and sustainable agriculture for economic growth and public health [8], innovative approaches like PFM are becoming increasingly essential for addressing the intricate challenges faced by China’s agricultural sector.

Weeds, known for their challenging management, are a major factor limiting crop production [9,10]. They compete intensely with crops for vital resources like water, nutrients, and light, leading to significant yield reductions [11]. Additionally, weeds can harbor various pests and diseases, further exacerbating their adverse impacts on agricultural ecosystems and productivity [12]. Conventional weed control methods, such as heavy reliance on herbicides, raise significant concerns about environmental pollution [13], herbicide resistance evolution [14], and food safety [15]. Therefore, integrating innovative weed management strategies, such as biodegradable plastic film mulching (BPFM), into crop production systems is crucial to enhance crop productivity and sustainability [16]. PFM itself acts as a physical barrier, reducing weed emergence and density, which, in turn, enhances crop growth and development [17].

However, a critical limitation of traditional PFM practices, employing non-degradable PE film, is the generation of persistent plastic residues in the soil [18]. This accumulation poses significant long-term risks to soil structure, health, and microbial activity, hindering nutrient cycling and ultimately threatening agricultural sustainability and environmental safety [19]. PE film residues in sugarcane fields exceed 250 kg/ha in intensive regions, complicating mechanized harvesting and increasing production costs [20]. Certified BPF offers a viable solution by providing comparable agronomic benefits while minimizing residual pollution [21]. Recent studies confirm that optimized BPF formulations can achieve >90% degradation efficiency within crop cycles without compromising yield [22].

Sugarcane stands as an important agricultural crop in China, and it accounts for 86.6% of the national sugar output [23]. Ensuring the safety of the national sugar supply is therefore intimately linked to the development of the sugarcane industry. Sugarcane yield is influenced by a complex interplay of factors, such as climate, soil fertility, variety selection, irrigation, fertilization, diseases, pests, and particularly, weeds [24]. The application of PFM to sugarcane cultivation has been shown to increase yields significantly, primarily by conserving soil moisture, elevating soil temperature in cooler periods, and suppressing weed pressure [25]. Specifically, the weed suppression effect of PFM is an effective method for promoting crop growth and development, and critically, the adoption of BPFM offers the potential to retain these benefits while addressing the residue pollution issue, thereby facilitating sustainable sugarcane agriculture [26]. This shift aims to ensure high yields while minimizing the adverse environmental impacts associated with conventional PE film mulching [27]. Thus, embracing environmentally friendly BPF as a substitute for conventional PE offers a promising pathway to mitigate potential harm from film residue on agricultural ecosystems and human health, potentially extend crop growth cycles under favorable conditions, and contribute to the overarching goal of ensuring sustainable development in the sugarcane industry.

Building upon this context, the present study explored the relationships among weed dynamics, sugarcane yield and various sugarcane agronomic characteristics under different film mulching treatments, specifically comparing conventional PE film, BPF, and no-mulch controls. We hypothesized the following: Both PE and BPFM would effectively suppress weed emergence and growth compared to bare soil; BPFM would achieve comparable weed suppression and yield enhancement benefits to conventional PE film; and consequently, BPF represents the most sustainable mulching option for meeting the growing demands of the sugarcane industry by balancing productivity and environmental protection. The objectives of this study were the following: to determine the optimal mulching conditions for maximizing sugarcane productivity; identify the major weed species and quantify their hazards during the critical sugarcane planting and early growth period; and based on weed dynamics and crop response, determine the optimal mulching period for attaining high sugarcane yield under BPF application.

## 2. Results

### 2.1. The Impact of Plastic Film Mulching on the Germination of Sugarcane Seedlings

**Figure 1:**
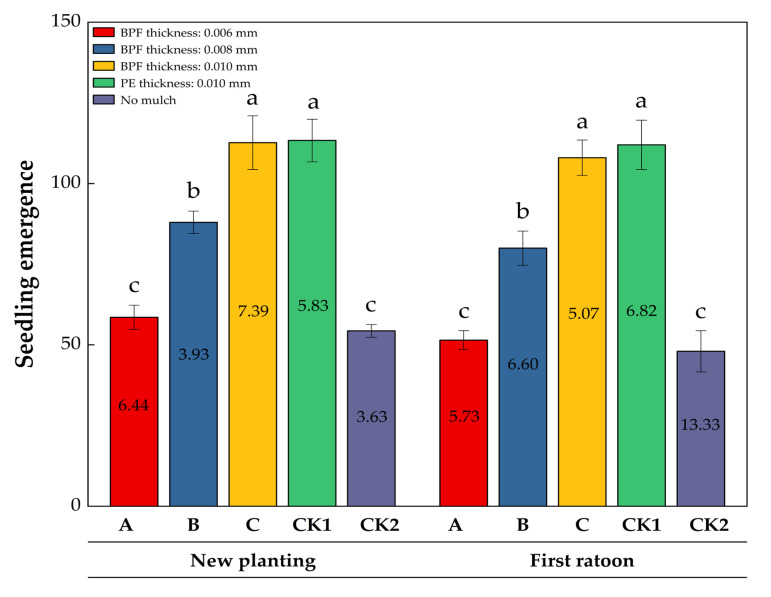
Effect of different film mulching treatments on sugarcane germination in new planting and the first ratoon season. The data presented are mean ± SD. Different lowercase letters (a, b, c) above the bars indicate significant differences among treatments at the *p* < 0.05 level (no significant difference indicated by the same letter). Numbers within the bars represent the coefficient of variation (*CV*) for seedling emergence rate under corresponding treatments and years. Data were analyzed using one-way analysis of variance (ANOVA) followed by Tukey’s honestly significant difference (HSD) test for multiple comparisons.

To investigate the thickness dependent effects of BPFM, three treatments (A: 0.006 mm, B: 0.008 mm, and C: 0.010 mm) were compared with two controls: a PE mulch (CK1, 0.010 mm, conventional practice) and non-mulched soil (CK2, baseline) (Figure 1). Film mulching enhanced sugarcane seedling emergence, and the germination rate was positively correlated with film thickness. Data on seedling emergence collected over two years (new planting and first ratoon) revealed significant variations. In new planting, treatments C (112.67) and CK1 (113.33) had the highest average seedling emergence, while CK2 was the lowest (54.33). Treatment B showed intermediate performance (88.00). This trend continued into the first ratoon, where CK1 (112.00) and C (108.00) maintained their advantage, while CK2 sharply declined to 48.00. The average seedling emergence for all treatments in the first ratoon was lower than in new planting. Each group’s average decline was approximately 8.2%, with CK2 showing the largest decrease (11.7%). This suggests a negative impact of interannual environmental changes on seedling establishment. In the first ratoon, treatment C achieved the lowest coefficient of variation (*CV*) (5.07%), indicating improved interannual stability. Conversely, CK2 exhibited the highest *CV* (13.33%), highlighting its unreliability under changing conditions. Overall, the *CV* increased by an average of 38% from new planting to first ratoon, emphasizing the role of annual fluctuations in data dispersion.

In summary, treatments C and CK1 consistently provided high seedling emergence rates, with C demonstrating enhanced stability in the first ratoon. The year effect significantly reduced seedling emergence rates and increased variability, particularly in control groups like CK2. These findings suggest that optimized treatments (such as C) can mitigate the annual uncertainty in seedling establishment.

### 2.2. Effect of Film Mulching on Weeds

**Figure 2:**
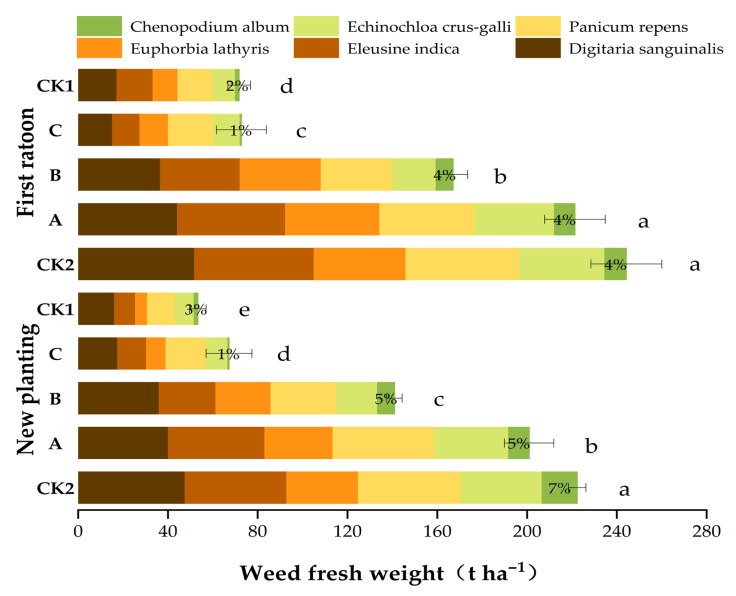
Effect of PFM on weeds. The data presented are mean ± SD. Different lowercase letters (a, b, c, d, e) above the bars indicate statistically significant differences among treatments at *p* < 0.05 (treatments sharing the same letter are not significantly different). Numerical values above the bars represent the proportion of broadleaf weeds in the weed community. Data were analyzed using one-way analysis of variance (ANOVA) followed by Tukey’s (HSD) test for multiple comparisons.

The practice of film mulching could control weeds in the field (Figure 2). Handling C consistently demonstrated superior weed suppression efficacy over two consecutive years, significantly reducing total weed biomass by approximately 70% relative to the untreated control (CK2). This treatment has emerged as a robust long-term strategy for integrated weed management, exhibiting pronounced effectiveness against resistant weed species such as *Digitaria sanguinalis*. The marked interannual consistency underscores its operational reliability under field conditions. Responses to individual weed species revealed distinct sensitivity patterns: *Chenopodium album* exhibited extreme susceptibility to both Handling C and the conventional herbicide control (CK1), resulting in near-complete biomass elimination. In contrast, *Panicum repens* and *Euphorbia lathyris* displayed inherent herbicide resistance; biomass reduction in these species was limited even under optimized Handling C application.

Five gramineous weeds, i.e., *Digitaria sanguinalis*, *Eleusine indica*, *Euphorbia lathyris*, *Panicum repens*, and *Echinochloa crus-galli*, were the most dominant weeds in the field, with gramineous weeds accounting for ≥93%. The incidence of broadleaf weeds was generally low in all treatments.

### 2.3. Effect of Film Mulching on Sugarcane Yield-Related Traits

**Figure 3:**
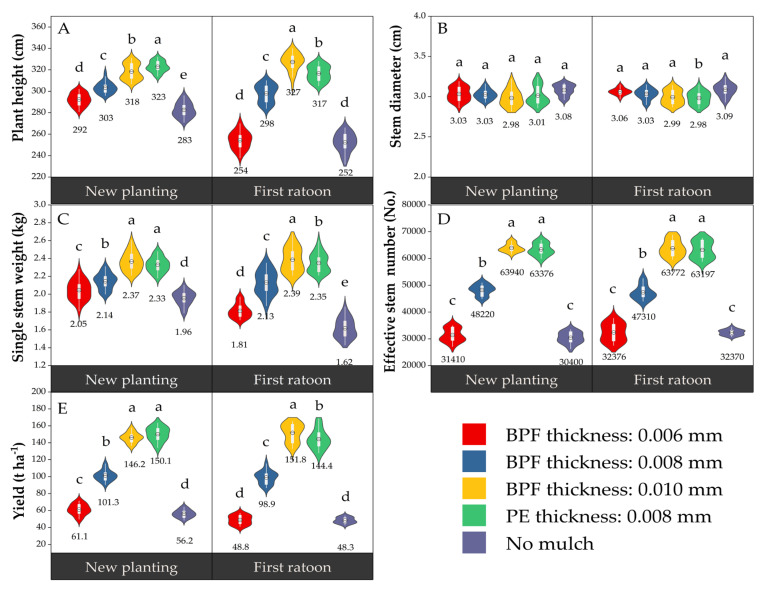
Effects of PFM on yield-related characteristics of sugarcane. The data values in each group figure represent the mean values of the corresponding treatments. Letters in top-left corners indicate: (**A**) Plant height, (**B**) Stem diameter, (**C**) Single stem weight, (**D**) Effective stem number, (**E**) Yield. Different lowercase letters (a, b, c, d, e) above the violins denote statistically significant differences among treatments at *p* < 0.05 (treatments sharing the same letter are not significantly different).

Mulching film significantly altered key yield-related components, with the exception of stem diameter (Figure 3). Significant differences were observed among treatments for plant height, single stem weight, effective stem number, and yield. Overall, treatments C and CK1 exhibited superior performance, while CK2 consistently ranked lowest. Treatment C increased the annual yield by 3.83% (+5.6 t ha^−1^ from new planting to first ratoon season), whereas Treatment CK1 decreased the annual yield by 3.80% (−5.7 t ha^−1^ over the same period). Notably, the perennial root system annual yield of Treatment C surpassed that of CK1 by 7.4 t ha^−1^.

### 2.4. Analysis of Year and Treatment Interaction Effects on Sugarcane Yield-Related Traits

The treatment had extremely significant effects on all yield-related traits (*p* < 0.01), indicating that treatment is the primary factor influencing sugarcane yield and associated agronomic characteristics (Table 1). Year also showed extremely significant effects on most yield-related traits (*p* < 0.001), except for stem diameter and effective stem number, suggesting that these two traits are more susceptible to annual environmental variations.

In the analysis of interaction effects (Year × Treatment), the interactions were found to be extremely significant (*p* < 0.01) for plant height, single-stem weight, and overall yield. However, no significant interaction effects (*p* > 0.05) were observed for seedling emergence, weed fresh weight, stem diameter, and effective stem number, indicating that these traits were less influenced by the combined effects of year and treatment.

### 2.5. Correlation Analysis of Measured Parameters

**Figure 4:**
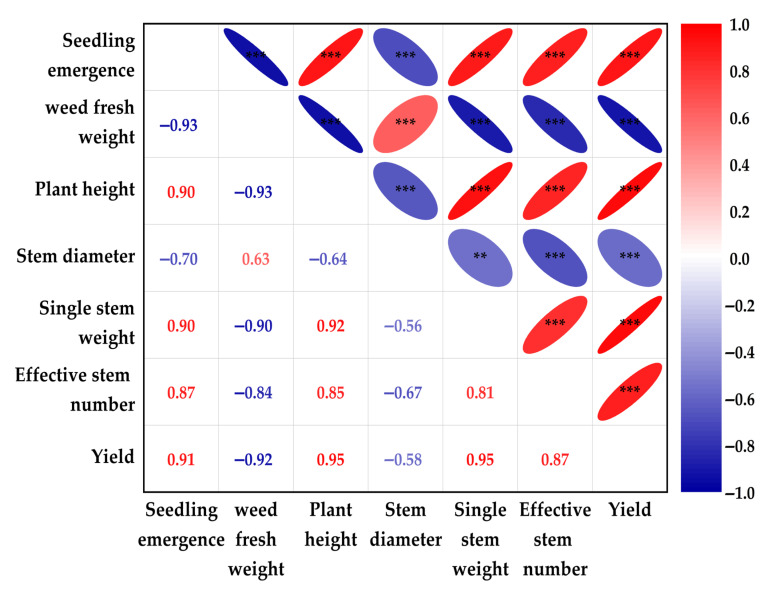
The correlations among the measured parameters under different film mulching treatments. In the correlation matrix, right-positioned red ellipses denote positive correlations and left-positioned blue ellipses indicate negative correlations, where both reduced ellipse size and intensified color saturation proportionally encode higher statistical significance of the correlation coefficients, with larger dimensions or lighter hues conversely representing lower significance levels. ** *p* < 0.01; *** *p* < 0.001 (Spearman’s rank correlation analysis).

The analysis demonstrates that yield exhibits strong positive correlations with key growth indicators—seedling emergence, plant height, single stem weight, and effective stem number (*r* = 0.87 to 0.91, *p* < 0.001) (Figure 4). Conversely, yield exhibited a strong negative correlation with weed biomass (*r* = −0.92, *p* < 0.001) and a weak negative correlation with stem diameter (*r* = −0.58, *p* < 0.01), highlighting weeds as a major yield-limiting factor. Critically, weed biomass significantly suppressed all growth metrics, showing strong negative correlations with seedling emergence, plant height, single stem weight, effective stem number, and yield (*r* = −0.84 to −0.93, *p* < 0.001), while weakly positively correlating with stem diameter (*r* = 0.63, *p* < 0.001). Furthermore, synergistic interactions were observed among seedling emergence, plant height, single stem weight, effective stem number, and yield, all strongly positively intercorrelated (*r* > 0.81, *p* < 0.001), highlighting their collective role in productivity.

### 2.6. Regression Analysis of Influencing Factors on Sugarcane Yield

Based on correlation analysis, a stepwise linear regression was performed with yield as the dependent variable (Table 2). Among the regression models evaluated under PFM conditions, Model 5 demonstrated the highest goodness-of-fit (*R*^2^ = 0.996), equivalent to Model 4. However, Model 5 exhibited superior statistical reliability, as all its independent variables contributed highly significantly (*p* < 0.001) with no multicollinearity issues (*VIF* < 5). In contrast, Model 4 and Model 3 showed inflated *VIF* values exceeding acceptable thresholds, compromising their stability and interpretability, despite partial statistical significance (e.g., plant height in Model 4). Consequently, Models 3 and 4 were excluded to ensure robustness.

Model 5 was identified as the optimal predictor (*R*^2^ = 0.996, F = 2148.133 ***, VIF < 5), revealing that sugarcane yield under PFM is predominantly driven by three traits: x_1_ (effective stems), x_2_ (plant height), and x_3_ (stem diameter).

The linear regression equation is as follows (Formula (1)):

(1) $$y\;=\;-296.116\;+\;0.821x_{1}\;+\;0.243x_{2}\;+\;0.061x_{3}$$

### 2.7. Logistic Fitting Analysis of the Effect of Film Mulching Time on Weeds and Sugarcane Yield

**Figure 5:**
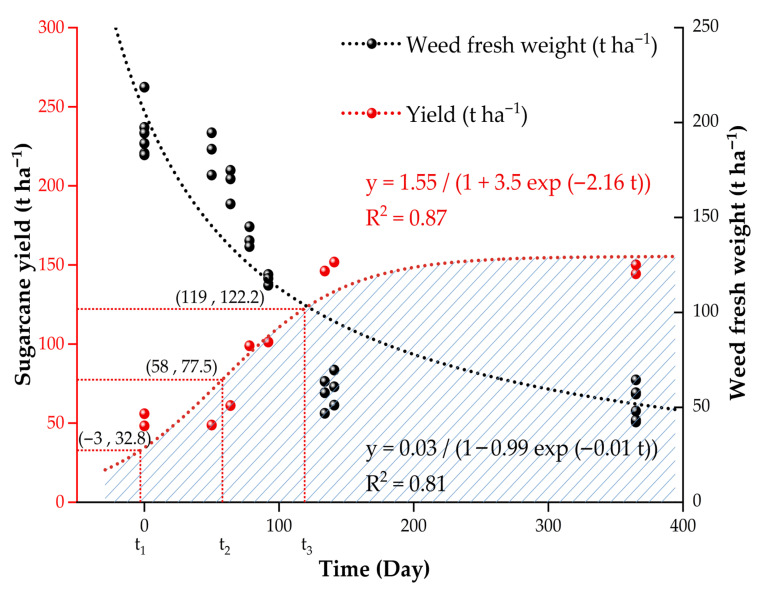
The logistic fitting curve for film mulching time against weed fresh weight and sugarcane yield. The logistic fitting curve is fitted by nonlinear regression analysis. *t*_1_, *t*_2_, and *t*_3_ predict the yield change law in each stage according to the first, second, and third derivatives.

The impact of film mulching time was fitted against sugarcane yield (Figure 5), and the equation of the curve is as follows (Formula (2)):

(2) $$y\;=\;\frac{1.55}{\left(1\;+\;3.5\;exp\;(-\;2.16\;t)\right)}$$

The curve had a good fitting effect, with *R*^2^ = 0.87. According to the law of yield change, the time inflection points were established based on the first derivative, the second derivative, and the third derivative. The low yield mulching period is <58 days, with a yield of 0–77.5 t ha^−1^. The medium yield mulching period is 58–119 days, with an output of 77.5–122.2 t ha^−1^. The high yield mulching period is >119 days, with a yield of >122.2 t ha^−1^.

The effect of the mulching time on weed fresh weight was fitted with a logistic function (Figure 5), and the equation is as follows (Formula (3)):

(3) $$y\;=\;\frac{0.03}{\left(1\;-\;0.99\;exp\;(-\;0.01\;t)\right)}$$

The fitting effect was good, and *R*^2^ = 0.81. The logistic curve of weeds and sugarcane yield showed that the overall performance based on weeds and yield was optimal after a mulching time of 119 days.

## 3. Discussion

Weeds present a major and persistent challenge to global crop productivity, causing substantial yield losses and demanding intensive management efforts. This study confirms PFM as a highly effective, non-chemical strategy for suppressing weeds in sugarcane cultivation. Demonstrating weed inhibition rates ranging from 9.5% to 73.2% and yield increases between 5.2% and 185.1% without the use of herbicides, PFM’s efficacy aligns with the yield benefits previously documented in mulched maize systems. The dominant weed type observed (≥93% cover) was gramineous weeds, mirroring infestations common in crops like rice, wheat, and corn, which underscores PFM’s particular relevance for controlling grass weeds specifically in sugarcane. Support for the physiological impact comes from metabolomic studies [28], confirming significant stress-induced shifts in the metabolic pathways of weed seeds developing under the mulch. Importantly, PFM aligns with organic farming principles by reducing reliance on synthetic herbicides and mitigating their associated environmental contamination and residue concerns.

Optimal film thickness balances functionality—including weed control and microclimate modulation—with mechanical resilience and cost-effectiveness. While 0.010 mm and 0.012 mm films are suitable for crops like cotton and corn [29], a thickness of 0.010 mm proves optimal for sugarcane. This specific thickness provides sufficient mechanical integrity to resist tearing during laying and field operations, alongside high light transmission essential for soil warming and promoting crop growth [30]. Critically, 0.010 mm BPF matched conventional polyethylene PE in weed control efficacy while eliminating the long-term plastic residue pollution risks associated with PE films.

Accumulated polyethylene PE residues detrimentally impact crop productivity globally [31,32], a stark contrast evident in our results: sugarcane mulched with biodegradable film BPF showed a +3.83% annual yield increase (+5.6 t ha^−1^), whereas PE-mulched sugarcane (CK1) exhibited a −3.80% annual yield decrease (−5.7 t ha^−1^). This decline stems from PE residues physically and chemically impairing sugarcane growth, including root and shoot development and nutrient/water uptake, underscoring the critical need to adopt BPF for sustainable production. Beyond boosting yield, BPF offers significant environmental benefits: it preserves soil structure by avoiding the ~12% reduction in soil macroporosity caused by PE, which hinders root function [33]; it drastically reduces microplastic accumulation, degrading into non-toxic oligomers, CO_2_, H_2_O, and biomass, resulting in a 92% reduction in soil microplastics compared to PE [34]; and it lowers the carbon footprint, with Life Cycle Assessment (LCA) indicating that BPF reduces sugarcane production carbon emission intensity by 17.3% [35].

Managing the degradation kinetics of biodegradable plastic film BPF is essential for maximizing its agronomic benefits while minimizing ecosystem risks [36]. Premature degradation compromises key functions such as weed suppression and microclimate control, negatively impacting crop yield, as demonstrated in cotton studies [37]. Our logistic regression analysis for sugarcane further confirms that maintaining BPF functional integrity for more than 119 days is critical for achieving high yield stability. Consequently, the BPF degradation rate must be carefully calibrated to align with the specific crop’s growth cycle and critical weed competition periods, ensuring the film remains intact throughout key developmental stages like crop establishment, tillering, and canopy closure. Post-harvest tillage, conducted under conducive soil conditions, facilitates the incorporation of film fragments and accelerates their biodegradation [38]. The integration of predictive degradation models, as highlighted in recent research [39], can significantly enhance this calibration process and overall BPF management strategies.

## 4. Materials and Methods

### 4.1. Experimental Site

**Figure 6:**
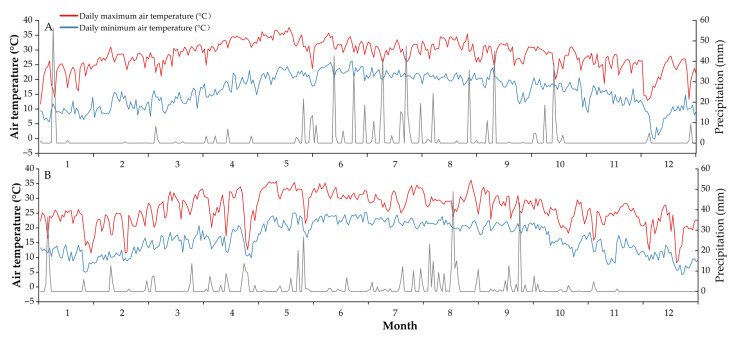
The daily maximum and minimum air temperature and daily precipitation during the experimental year 2019 (**A**) and 2020 (**B**).

A two-year field experiment was set up at the Second Scientific Research Base of the Sugarcane Research Institute of the Yunnan Academy of Agricultural Sciences (23°44′05″ N, 103°16′30″ E; Elevation: 1100 m) on December 1, 2018. The previous crop was sugarcane. The soil was latosol, with the basic properties of topsoil (0–20 cm) as follows: pH 6.2, organic matter 23.0 g kg^−1^, total nitrogen 1.0 g kg^−1^, alkali-hydrolysable nitrogen 65.8 mg kg^−1^, total phosphorus 0.9 g kg^−1^, available phosphorus 35.2 mg kg^−1^, total potassium 13.8 g kg^−1^, and available potassium 86.6 mg kg^−1^. In addition, the rainfall, daily maximum temperature and daily minimum temperature during the experiment were recorded by the Yunnan Meteorological Center (Figure 6).

### 4.2. Experimental Materials

The sugarcane variety ‘YZ081609’ was used in the experiment. This variety is characterized by high drought resistance, defoliation, strong ratooning, early maturing, high yield and high sugar content [40]. The mulch films used were 0.006 mm thick (A), 0.008 mm thick (B), and 0.010 mm thick (C) BPF provided by Shandong Tianzhuang Environmental Protection Technology Co., Ltd. (Jinan, China), and 0.010 mm thick PE (CK1) provided by the Sugarcane Research Institute, Yunnan Academy of Agricultural Sciences (Kaiyuan, China). All films were 1.5 m wide.

### 4.3. Experimental Design and Measurements

A randomized complete block design was used in the field experiment with five treatments, including 0.006 mm thick (A), 0.008 mm thick (B), and 0.010 mm thick (C) BPF, 0.010 mm thick PE (CK1), and a no mulching control (CK2). Each treatment had three replicates, yielding a total of 15 plots. Each plot had an area of 36 m^2^ with six planting rows, and the row spacing and length were 1.0 m and 6.0 m, respectively. Yield and growth parameters were measured only from the four central rows of each plot, excluding edge rows (Row 1 and Row 6). Sugarcane was planted at a density of 120,000 buds ha^−1^ (equivalent to 12 buds per square meter) in the new planting season. The same rates of chemical fertilizers were applied in both new planting and the first ratoon seasons with N 577.8 kg ha^−1^, P_2_O_5_ 266.7 kg ha^−1^, and K_2_O 155.6 kg ha^−1^.

During the sugarcane whole growth period, no herbicides were applied, and fertilizers were applied once before mulching in each cropping season. In a seedling emergence survey, data were collected from a total area of 75 square meters across three replicate plots. Within each plot, five randomly selected sampling units were assessed, with each unit representing a discrete 5-square-meter area. Edge areas more than 1 m from the field boundary were excluded to avoid edge effects. The sequence of field operations for both newly planted and first ratoon seasons was meticulously documented (Table 3 and Table 4), including the timing of mulch film application for each treatment and the time nodes from the laying of biodegradable mulch films of different thicknesses until the degradation rate reached over 90% in the farmland environment [41].

### 4.4. Statistical Analysis

All the data were sorted by WPS Office (Kingsoft). IBM SPSS Statistics (version 24.0; IBM Corp., Armonk, NY, USA) software was applied for statistical analysis. Tukey’s test was used to compare the germination rate between treatments and Spearman correlation analysis was used to analyze the correlations between measured parameters. Multiple linear regression analysis was conducted by progressive analysis and stepwise analysis, while the logistic growth curve regression model was fitted by nonlinear regression. Origin 2021 (OriginLab Corp., Northampton, MA, USA) was used to plot graphs.

The key agronomic parameters are calculated according to the definitions provided in Table 5.

The multiple linear regression model is based on the linear correlation between the dependent variable (*y*) and two or more independent variables (*x*), and thus multiple independent variables are used to jointly predict the change in the dependent variable [42]. The regression equation is as follows (Formula (4)):

(4) $$y\;=\;\beta_{0}\;+\;\beta_{1}x_{1}\;+\;\beta_{2}x_{2}\;+\;\ldots \;\beta_{n}x_{n}$$

where β_0_ represents the non-standardized coefficient constant, β_i_ (i = 1, 2, … n) represents the non-standardized coefficient of the independent variable, and x_i_ (i = 1, 2, … n) represents the independent variable.

The logistic growth curve regression model is established based on the logistic function to determine a highly fitting model [43]. The equation is as follows (Formula (5)):

(5) $$y\;=\;\frac{k}{\left(1\;+\;{ae}^{-bt}\right)}$$

Using the first-order derivative, second-order derivative, and third-order derivative of this equation, the yield change in each stage was predicted. This model divides the growth period into three time inflection points: t_1_, t_2_, and t_3_. The equation is as follows (Formula (6)):

(6) $$t_{1}\;=\;\frac{\left(lna\;-\;1.317\right)}{b};\;t_{2}\;=\;\frac{lna}{b};\;t_{3}\;=\;\;\frac{\left(lna\;+\;1.317\right)}{b}$$

Here, t is the independent variable; y is the cumulative yield; a, b, and k are morphological parameters, and e is the exponential function. The low yield period occurs before t_2_, the medium yield period ranges from t_2_ to t_3_, and the high yield period is after t_3_.

**Table 5:** The following formulas and methods are used for data analysis.

Data	Formulas and Methods
Germination Rate	(Number of sprouted seedlings/Total number of seedlings planted) × 100% [44].
Weed Fresh Weight	Weeds in five 1 m^2^ quadrats per plot were collected randomly using the “s” sampling method, cut at ground level (stubble height ≤2 cm) to exclude roots. Fresh weed weight measurements were conducted within 2 h post-harvest after removing extraneous matter and gently brushing off adhering soil, using an XK3190-A27E electronic weighing indicator (measurement range: 0–30 kg, readability: 0.1 g) [44].
Weed Control Effect	Weed Inhibition Percentage (%) = [ CK_2_ Average Weed Fresh Weight/(CK_2_ Average Weed Fresh Weight–Treated Group Average Weed Fresh Weight)] × 100% [45].
Plant Height	Was measured; 10 representative sugarcane plants were selected from each plot before harvest, and the lengths from the base of the stem to the highest visible hypertrophy zone were measured [46].
Stem Diameter	Was measured before harvest, 10 representative sugarcane plants were selected from each plot, and a caliper was used to measure the stem diameter at the middle internode on the sugarcane stem in the direction of the bud [47].
Effective Stem Number	Sugarcane effective stems with a length >1 m (excluding dead stems) [44].
Yield	Fresh weight of sugarcane stems in a 12 m^2^ area of the plot at harvest [44].

## 5. Conclusions

This study establishes that biodegradable film mulching (0.010 mm thickness) optimizes sugarcane yield and weed management. Key findings indicate the following: BPF mulch enhances seedling emergence stability (*CV* = 5.07% in the first ratoon) and suppresses dominant gramineous weeds by 70%, demonstrating superior performance over non-biodegradable PE mulch in perennial yield (yield change: BPF + 3.83% vs. PE − 3.80%); Yield is primarily driven by effective stem number, single stem weight, and plant height (Model 5, *R*^2^ = 0.996), with weed biomass as the major limiting factor (*r* = −0.92); A mulching duration >119 days is critical for high-yield phases (>122.2 t ha^−1^) and effective weed control, whereas durations <58 days result in significant yield losses; The synergy between film mulching and interannual environmental variation necessitates tailored management, particularly for ratoon crops. Thus, 0.010 mm biodegradable film is recommended as a sustainable solution to enhance productivity while mitigating plastic residue pollution.

## Figures and Tables

**Table 1:** Analysis of variance for treatment and year effects on sugarcane traits.

Variable	Year (*F/p*)	Treatment (*F/p*)	Year × Treatment (*F*/*p*)	DF (Year)	DF (Treatment)	DF (Year × Treatment)
Seedling emergence	25.519 ***	827.103 ***	0.498 ns	1	4	4
Weed fresh weight	28.568 ***	415.916 ***	1.036 ns	1	4	4
Plant height	49.638 ***	129.603 ***	18.530 ***	1	4	4
Stem diameter	0.079 ns	6.046 **	0.465 ns	1	4	4
Single stem weight	26.130 ***	118.431 ***	12.425 ***	1	4	4
Effective stem number	0.834 ns	1510.100 ***	1.902 ns	1	4	4
Yield	10.385 ***	950.905 ***	4.583 **	1	4	4

F-values from between-subject effects tests are reported for univariate analyses: *** *p* < 0.001; ** *p* < 0.01; ns: not significant.

**Table 2:** Regression analysis of influencing factors on sugarcane yield under PFM.

Dependent Variable	Yield							
Predictive Variable		Effective Stem Number	Single Stem Weight	Plant Height	Stem Diameter	*R* ^2^	*F/p*	*β*_0_
Model 1	*β*	0.990				0.980	1452.399 ***	−39.575
	*T*	38.110 ***						
	*VIF*	1.000						
Model 2	*β*	0.779	0.241			0.994	2283.336 ***	−95.993
	*T*	25.048 ***	7.738 ***					
	*VIF*	4.440	4.440					
Model 3	*β*	0.769	0.131	0.122		0.995	1783.611 ***	−114.935
	*T*	26.491 ***	2.415 *	2.367 *				
	*VIF*	4.531	15.898	14.346				
Model 4	*β*	0.802	0.082	0.175	0.051	0.996	1706.543 ***	−263.567
	*T*	28.426 ***	1.591 ns	3.544 **	2.851 **			
	*VIF*	5.455	17.989	16.678	2.187			
Model 5	*β*	0.821		0.243	0.061	0.996	2148.133 ***	−296.116
	*T*	31.368 ***		9.627 ***	3.508 ***			
	*VIF*	4.438		4.122	1.933			

Model 1: Effective stem number; Model 2: Effective stem number, single stem weight; Model 3: Effective stem number, single stem weight, plant height; Model 4: Effective stem number, single stem weight, plant height, stem diameter; Model 5: Effective stem number, plant height, stem diameter. The analysis employed stepwise linear regression. *β*_0_ represents the non-standardized coefficient constant; *β* represents the non-standardized coefficient of the independent variable; *T* represents the significance test of the regression coefficient; *VIF* represents the variance expansion factor value; *R*^2^ represents the adjusted fitting coefficient; and *F* represents the significance test of the regression equation. * *p* < 0.05; ** *p* < 0.01; *** *p* < 0.001; ns: not significant.

**Table 3:** Chronological sequence of field operations for new planting and the first ratoon season.

Operation	New Planting Cycle	Days After Planting	First Ratoon CYCLE	Days After Ratooning
Mulching Application	2 December 2018	Day 0	3 January 2020	Day 0
Seedling Emergence	6 March 2019	Day 95	3 April 2020	Day 91
Weed Assessment	17 May 2019	Day 167	19 June 2020	Day 169
Yield Measurement	6 December 2019	Day 370	7 January 2021	Day 371

**Table 4:** The covering time of different mulching treatments during new planting and the first ratoon season.

Cropping Season	Mulching Method	Covering Time (Days)
New planting	A (0.006 mm BPF)	64
	B (0.008 mm BPF)	92
	C (0.010 mm BPF)	134
	CK1 (0.010 mm PE)	365
	CK2 (no mulching)	0
First ratoon	A (0.006 mm BPF)	50
	B (0.008 mm BPF)	78
	C (0.010 mm BPF)	141
	CK1 (0.010 mm PE)	365
	CK2 (no mulching)	0

## Data Availability

The raw data supporting the conclusions of this article will be made. available by the authors on request.
